# Claudin Family of Proteins and Cancer: An Overview

**DOI:** 10.1155/2010/541957

**Published:** 2010-07-08

**Authors:** Amar B. Singh, Ashok Sharma, Punita Dhawan

**Affiliations:** ^1^Department of Surgery, Vanderbilt University Medical Center, Nashville, TN37232, USA; ^2^Cancer Biology, Vanderbilt University Medical Center, Nashville, TN37232, USA

## Abstract

Tight junctions are the apical cell-cell adhesion that regulate paracellular permeability and are critical for epithelial cell polarity. Molecular architecture of tight junction has been studied extensively, which has confirmed that claudin family of proteins is integral component of tight junction. Loss of cell-cell adhesion is central to the cellular transformation and acquisition of metastatic potential; however, the role of claudin family of proteins play in a series of pathophysiological events, including human carcinoma development, is only now beginning to be understood. Several claudin mouse knockout models have been generated and the diversity of phenotypes observed clearly demonstrates their important roles in the maintenance of tissue integrity in various organs and suggest that claudins also participate in cellular contexts other than tight junctions. The mechanisms of claudin regulation and their exact roles in normal physiology and disease are being elucidated, but much work remains to be done. In this review, we have discussed the conceptual framework concerning claudins and their potential implication in cancer. We predict that next several years will likely witness a boom in our understanding of the potential role of claudins in the regulation of tumorigenesis, which may, in turn, provide new approaches for the targeted therapy.

## 1. Introduction

Genetic alterations in various genes responsible for the maintenance of the normal epithelial phenotype has come forward as a major cause for the deregulation of normal epithelial physiology however, it is well established that genetic mutations are correlated with various environmental stimuli. In addition, direct exposure to various environmental carcinogens is considered one of the most plausible sources of inducing neoplasia. In general, mammalian body is very selective in its absorptive behavior which is regulated by size as well as charge of the molecules which body is being exposed. Tight junctions, the most apical cell-cell adhesion, owing to their cellular location are responsible for this selection and any qualitative or quantitative deregulation of the TJ characteristics could potentially change the normal equilibrium maintained, resulting in abnormal cellular physiology. Also, the normal regulation of growth factor receptor activation due to differential distribution of the receptor and the respective ligands can be compromised due to irregular tight junctions [1]. Disruption of tight junction barrier function and changes in permeability properties have been shown to be associated with a number of pathologic conditions such as kidney disorders, inflammatory bowel disease, pulmonary edema, diarrhea, and jaundice [2–5]. Proper cell-cell and cell-extracellular matrix interactions are essential for normal functioning of an epithelial cell and it is known that various cell adhesion proteins such as E-cadherin, *β*-catenin, or *β*1-inetgrin perform functions different than their normal cell adhesion function upon loss of normal cell-cell or cell-ECM adhesions [6]. A similar hypothesis could be postulated for the proteins forming the tight junctions, which could probably play a central role in the neoplastic process via coupling of the extracellular milieu to intracellular signaling pathways and the cytoskeleton. In this regard, ZO-1, a tight junction protein, binds with the Y-box transcription factor ZONAB that has been shown to increase cell proliferation and decrease differentiation [7]. Recently, Symplekin, yet another transcription factor, was shown to increase tumorigenicity of the colon cancer cells through the upregulation of claudin-2 and ZONAB [8]. Importantly, ZO-1 and ZONAB are localized at the tight junction in differentiated and polarized epithelial cells while translocate to the cell cytoplasm/nucleus in proliferative or dedifferentiated cells [9]. In this paper, we will summarize the current knowledge regarding the role of tight junction with specific emphasis upon the claudin family of proteins in cancer and potential cause and effect association between the expression of specific claudin family members with tumor growth and progression.

## 2. Tight Junction and Tumorigenesis

Tight junctions (TJs) are the most apical intercellular junctions in epithelial and endothelial cells. The two major functions defined for tight junctions are the regulation of paracellular permeability through its barrier function and maintenance of the cell polarity through the fence function [10, 11]. These considerations of polarity, compartmentation, and barrier function are the underpinnings of a fascinating development in biomedicine. The fence function of tight junction helps in maintaining cell polarity, thus preventing intermixing of molecules in the apical membrane with those in the lateral membrane. There are certain times in any area of scientific research where one can witness a new concept taking shape and gaining acceptance. The involvement of epithelial barrier breakdown in the development of epithelial neoplasia is such a concept at present that is gaining acceptance and importance, although it is important to mention that the “roots” of this concept go back many years. The function of tight junction that is deeply involved in cancer cell biology is epithelial paracellular permeability and the loss of cell polarity [12, 13]. 

The concept of epithelial barrier breakdown involves the three mutually interrelated elements that have key consideration in neoplastic growth and development: (i) as a result of cell polarity, functional growth factor receptors are normally situated on the basal-lateral cell surface facing interstitial fluid and the bloodstream; (ii) growth factor proteins (the ligands for these receptors) are frequently compartmentalized at very high concentrations in luminal fluids within epithelial tissues; and (iii) early in the process of neoplasia, “distortions” occur in TJs such that relatively large solutes may pass across epithelial barriers that normally restrict their movement, a phenomenon one might call “lesional leak.” For example, in colorectal cancer, expression of claudin-2 that has been correlated with epithelial permeability increases while expression of claudin-1 or 7 that are correlated with increased TER is either mislocalized or is decreased [14, 15]. Thus, the concept has developed that TJ disruption in premalignant neoplastic tissue can increase the likelihood that it will develop into a frank carcinoma because of the continuous stimulation of cell division of initiated (premalignant) cells that follows breakdown of the natural barrier between growth factors and their receptors. 

Studies have shown that the epithelial tight junctions are dynamic structures and are subject to modulation during epithelial tissue remodeling [16], wound repair [17], inflammation [18], and transformation into tumors [19].  The association of abnormal TJ function and epithelial tumor development has been suggested by earlier studies showing alterations in the TJ structures of epithelial cancers [20]. *In vitro* studies using epithelial cell lines demonstrated that monolayers can be transformed into multilayered polyp like structures by oncogenes, such as K-*ras* [21], or by phorbol ester tumor promoters [22]. Epithelial multilayering was associated with increased TJ permeability [22, 23], activation of protein kinase C-*α*  [24] and phosphorylation of TJ proteins [25].

## 3. Claudins: Tight Junction Integral proteins

**Figure 1:**
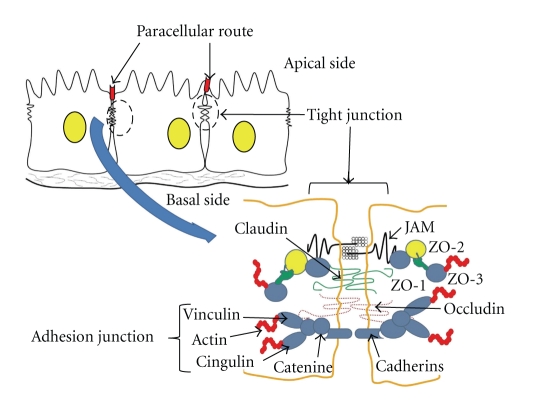
Schematic presentation of tight junction location between the epithelial cells and paracellular transport. Lower part represents tight junction strands and interaction of their major components.

Tight junctions are complex cellular entities and have always been understudied especially because of the lack of the precise knowledge of the proteins constituting them and also due to the difficulties associated with establishing *in vivo* or *in vitro *models to determine the true functional characteristics associated with these proteins. Although multiple proteins with diverse biological functions including tumor suppressors such as APC, PTEN, or cell polarity proteins such as Par-3, aPKC*λ* are localized at the tight junction location, it was only in the late 1980s that biochemical and immunolocalization studies identified the 225 kDa protein zonula occludens-1 (Z0-1) as the first polypeptide exclusively associated with the TJ [26]. ZO-2 and ZO-3, which are highly related to ZO-1, were identified later [27–29]. However, genetic manipulation studies suggested that the ZO-family of proteins, although associated with TJ are not the TJ integral proteins. Immunolocalization by both light and electron microscopy further revealed that all three known ZOs (ZO-1, ZO-2, and ZO-3) are located exclusively at the cytoplasmic surface of TJs in the immediate vicinity of the plasma membrane and not in the plasma membrane. Since then, a number of integral membrane proteins associated with the TJ have been identified during recent years including occludin [30], junctional adhesion molecule (JAM) [31], and the claudin family of proteins, which consists of at least 24 members [32] (Figure 1). The JAMs are immunoglobulin (Ig)-like single-span transmembrane molecules and mediate Ca^2+^-independent adhesion. They are concentrated at TJs as well as AJs, not only in polarized epithelial and endothelial cells but also in hematopoietic cells of all lineages [33]. These proteins can form homodimers or heterodimers to produce paired strands between adjacent cells, thereby determining the characteristic permeability properties of different epithelial tissues [34]. Occludin with four transmembrane domains was identified as the first TJ-specific integral membrane protein. However, occludin-deficient visceral endoderm cells still bore a well-developed network of TJ strands, pointing to the existence of as-yet-unidentified TJ-specific integral membrane proteins [35]. 

**Figure 2:**
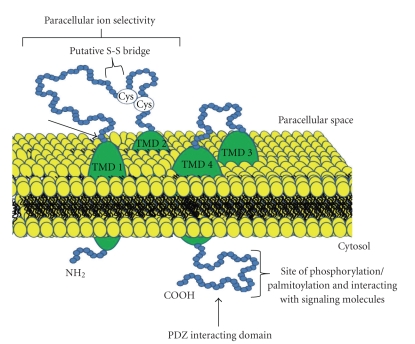
Schematic representation of structure of claudins. Claudins are transmembrane proteins with domains 1 to 4 (TMD-1, TMD-2, TMD-3, and TMD-4) and extracellular loops represent promising target for therapy. The –COOH terminal of claudins contains PDZ-binding domain which undergoes posttranscriptional modification that is important for signal transduction.

Using the same liver fraction employed to identify occludin, and by means of a sucrose step gradient, a single 22 kDa band was discovered as a putative novel TJ integral protein. Peptide sequencing revealed two proteins in this band that were subsequently named claudin 1 and 2 [32]. The name claudin derives from the Latin word “claudere” which means to close. Now, outcome of multiple studies since the initial discovery of claudin-1 and -2, has established that the claudin family of proteins are the major integral membrane proteins forming the backbone of tight junctions [32, 36, 37]. The claudin family consists of 24 known transmembrane proteins exhibiting distinct tissue- and development-specific distribution patterns [37, 38]. They are detected in both epithelial and endothelial cells and form a complex with occludin and/or JAMs [32, 39]. Claudins encode 20–27 kDa proteins with four transmembrane domains, two extracellular loops where the first one is significantly longer than the second one, and a short carboxyl intracellular tail (Figure 2). The last amino acids of this tail are highly conserved within the family and constitute PDZ binding motifs: claudins 1–9 and 17 S/TYV, claudins 10 and 15 AYV, claudin 11 AHV, claudin 12 HTT, claudin 13 LDV, claudins 14, 18 and 20 DYV, claudin 16 TRV, and claudin 19 DRV. Through these motifs, claudins are linked to the TJ PDZ containing proteins ZO-1, ZO-2, ZO-3 [40], PATJ [41] and MUPP1 [42]. A number of other cytosolic and nuclear proteins which includes regulatory proteins Rab3b, Rab13, tumor suppressors like PTEN, transcription factors like ZONAB, and HuASH1 have also been shown to interact directly or indirectly with tight junction complex [9, 43–45]. These interactions suggest that tight junctions, in addition to acting as barriers to paracellular flow of solutes, may play an important role in regulating other cell functions, such as proliferation and tumor suppression. For example, mutation in *CLDN14* leads to nonsyndromic recessive deafness [46] and the mutated *CLDN16* gene has been associated with hereditary hypomagnesemia [47]. Mice lacking claudin11 (also known as Occludin Sertoli Protein) have demonstrated the absence of TJ strands in myelin sheets of oligodendrocytes and Sertoli cells in the testis [48]. They show male sterility as well as delayed axonal conduction rates in the central nervous system. However, emerging details from a boom of studies related with the claudins in cancer have implicated claudin family members in a wide range of human cancer and in a tissue specific manner.

## 4. Claudins and Cancer

Since their discovery, literature regarding the status of claudins in various cancers is constantly expanding, and in contrast to the general thought that claudins expression would decrease during tumorigenesis as tight junctions are lost during cellular transformation, claudins expression seems to change in a tissue specific manner. Tan et al. [49] have shown that the expression and distribution of claudin-1 is associated with cell dissociation status in pancreatic cancer cells through mitogen-activated protein kinase 2 activation. By contrast, claudin-7 has been found to be decreased in invasive ductal carcinomas [50], head and neck cancer [51] and metastatic breast cancer [52]. On the other hand, Claudin-3 and -4 are frequently elevated in various cancers including pancreatic ductal adenocarcinoma, prostate, uterine, ovarian cancer [53] and breast cancer [54] while hepatocellular and renal carcinomas expressed lower levels of claudins-4 and -5 [55]. While, lower expression of claudin-2 was also seen in breast and prostatic carcinomas, expressions of claudin-1 and claudin-7 that were undetectable in normal cervical squamous epithelium increased in the cervical neoplasia [55, 56]. Intriguingly, recent studies have shown that expression of certain claudins especially claudin-1 and claudin-4 increases during metastasis and genetic inhibition of their expression has profound effect on the metastatic abilities of cancer cells though in a tissue specific fashion [57–59]. In Table 1, we have summarized the status of the expression of claudin family members in different cancer types. Intuitively, the mechanism by which decreased claudin expression might lead to the compromised TJ function and, thus, neoplasia is easy to comprehend, but how increased claudin expression contributes to neoplastic progression, as described here and by others, is less clear. One plausible mechanism is that upregulation or aberrant tissue expression of certain claudins may contribute to neoplasia by directly altering TJ structure and function. Furthermore, it is postulated that claudins may also affect cell signaling pathways. Claudin proteins are likely involved in signaling pathways via binding domains to ZO-1 at their carboxyl terminus [40]. Cell-cell adhesion proteins are known to play important role in cellular transformation when displaced from their normal membrane localization and could serve as oncogenic molecule. The best studied molecules is *β*-catenin, which although serve as cell-cell adhesion molecules when expressed in its normal cellular localization, *β*-catenin becomes oncogenic [60]. A similar functional heterogeneity could be postulated for claudins however further studies are needed to support such a notion. 

In this regard, we have recently demonstrated biological significance of altered claudin-1 expression in colon cancer cells. An increase in claudin-1 expression was observed in human primary colon carcinoma and metastasis samples and in the cell lines derived from primary and metastatic tumors as compared to their normal counterparts [59]. An important finding of our study was the nuclear localization of claudin-1 in a significant subset of colon cancer samples, particularly among the subset of liver metastatic lesions. Nuclear localization of several cell junction proteins (*β*-catenin, ZO-1, ZO-2) is known to be correlated with oncogenic transformation and cell proliferation [61, 62]. As mentioned above, *β*-Catenin plays a well-characterized dual role in cell adhesion (membrane localized) and in signal transduction (cytoplasmic and nuclear) leading to the epithelial cell transformation. Further, mutants of the TJ protein ZO-1 that no longer localize at the plasma membrane induce a dramatic epithelial-mesenchymal transition (EMT) of Madin-Darby canine kidney I cells [63]. Similarly, genetic manipulations of claudin-1 expression in colon cancer cell lines induced changes in cellular phenotype, with structural and functional changes in markers of epithelial-mesenchymal transition (EMT) and had significant effects upon the growth of xenografted tumors and metastasis in athymic mice. Notably, regulation of E-cadherin expression and *β*-catenin/Tcf signaling emerged as one of the potential mechanism underlying claudin-1– dependent changes and thereby suggested complex interplay between different cell-cell adhesion molecules [59]. There is accumulating evidence that the regulation of gene expression of tight junction proteins by the Wnt signaling pathway is part of a mechanism essential for the differentiation of epithelial cells, which is imbalanced in oncogenic transformation. Moreover, Wnt dependent signal transduction may be one way to influence barrier function which is essentially determined by the epithelial tight junctions. During recent years, a number of components found in junctional complexes of polarized epithelial cells have been shown to have signaling functions involved in cell growth and differentiation [64]. Activation of the Wnt pathway leads to the stabilization of *β*-catenin, which subsequently translocates into the cell nucleus and regulates gene expression in association with the lymphoid enhancer factor (LEF)/T-cell factor (TCF) family of transcription factors [65]. LEF/TCF are nuclear effectors of the Wingless (Wg)/Wnt signaling pathway, which is involved in the regulation of cell fate, differentiation, and polarization [66, 67]. Mutations in the gene for the adenomatous polyposis coli (APC) tumor suppressor protein stabilizes *β*-catenin and are supposed to be crucial events in oncogenic transformation of intestinal epithelial cells, which may develop into adenomas and carcinomas [68]. Expression of specific claudin family members can be regulated by Wnt signaling pathway. Claudin-1 and claudin-2 are shown to be target genes regulated by *β*-catenin signaling [69, 70]. Not only did expression of claudin-1 decreased significantly in response to the reduction of intracellular *β*-catenin by adenovirus mediated transfer of wild-type APC into the APC-deficient colon cancer cells, but also two putative Tcf4 binding elements in the 5' flanking region of claudin-1 were confirmed to be responsible for activating its transcription [69]. Further, nuclear effectors of the Wnt signaling pathway bind directly to the claudin-2 promoter region and thereby enhance claudin-2 promoter activity. They further demonstrated a crosstalk between the Wnt signaling pathway and Cdx related transcriptional activation with regard to claudin-2 promoter-mediated gene expression [70]. This suggests that Wnt signaling directly regulates the claudin-2 promoter via the LEF-1/*β*-catenin complex and indirectly enhances claudin-2 gene expression by transcription activation of Cdx1. Importantly, gene expression of another component of the tight junction complex, ZO-1, was suppressed after transient expression of *β*-catenin into human colonic cancer cell lines with low endogenous *β*-catenin, which is suggested to contribute to a loss of epithelial polarization in neoplastic cells [70]. Further, mutation of the *APC *gene (thus, *β*-catenin activation and nuclear translocation) is present in majority of the human colorectal carcinomas [71]. It is further interesting that colon cancer cells that expressed claudin-1 (HT29, SW480, and SW620) all harbor mutations in *APC* and have activated *β*-catenin/Tcf signaling. By contrast, RIE and HCT116 cells express wild-type *APC* [72], and neither cell line expresses detectable levels of claudin-1, and thus indicated that APC protein can regulate claudin-1 expression in *β*-catenin/Tcf dependent/independent manner. Similar dependence of claudin-1 expression in colon cancer cells upon APC and *β*-catenin signaling was also shown by others [69]. Metastasis is a complex phenomenon that requires a number of specific steps such as decreased adhesion, increased motility and invasion, proteolysis, and resistance to apoptosis [73]. Claudins expression increase the migration/motility as shown by both Boyden chamber and wound-healing assays [59, 74]. Claudin-5 promotes processing of pro-MMP-2 by MT1-MMP. Expression of claudin-5 not only replaced TIMP-2 in pro-MMP-2 activation by MT1-MMP but also promoted activation of pro-MMP-2 mediated by all MT-MMPs and MT1-MMP mutants lacking the transmembrane domain (DeltaMT1-MMP) [75]. Stimulation of MT-MMP-mediated proMMP-2 activation is also reported with other claudin family members including claudin-1, -2, and -3 [59, 75]. Amino acid substitutions or deletions in the ectodomain of claudin-1 abolished this stimulatory effect and direct interaction of claudin-1 with MT1-MMP and MMP-2 was demonstrated using immunoprecipitation. MT1-MMP was colocalized with claudin-1 not only at cell-cell borders, but also at other parts of the cell [75]. Thus it appears that interaction of MMP with claudins might play an important role in tumorigenesis, invasion and metastasis mediated by claudin expression. In our studies, we observed that overexpression of claudin-1 in colon cancer cells increased activity of both MMP-2 and MMP-9 while inhibition of claudin-1 resulted in a significant decrease in MMP-9 activity [59]. Similarly, overexpression of claudin-3 or 4 in ovarian epithelial cells increased matrix metalloproteinase-2 (MMP-2) activity [57]. 

**Figure 3:**
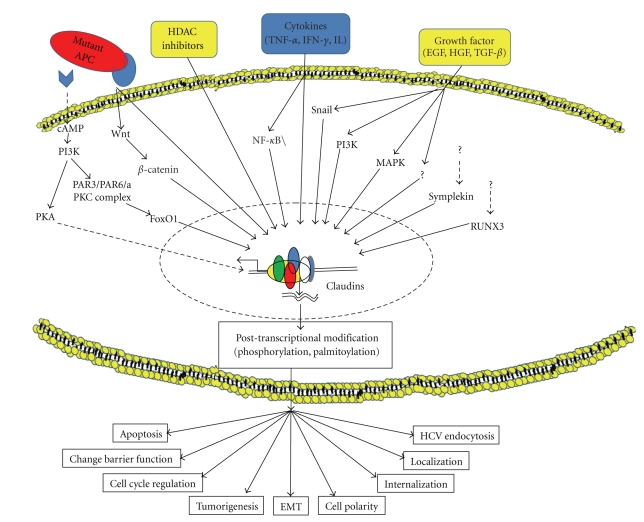
Schematic representation of multiple mechanisms implicated in the regulation of the expression and function of claudins. Broken lines indicate indirect pathways and solid lines represent direct pathways. Abbreviations: HDAC-Histone deacetylase; MAPKs- mitogen-activated protein kinases; RUNX3—Runt-related transcription factor 3; FOXO1—Forkhead box O-1; PAR3/PAR6-Partitioning defective; PI3K- Phosphoinositide 3-kinases; NF-*κ*B - nuclear factor kappa-light-chain-enhancer of activated B cells.

Claudin expression and functions are regulated at multiple levels and by diverse mechanisms [64, 76]. Delocalization of claudins from membrane appears to be common among the transformed cells [59, 77]. Constitutive activation of Ras or Ras-mediated signaling pathway/s is one of the initial steps during tumorigenesis that is causatively associated with neoplastic transformation. In Ha-Ras overexpressing MDCK cells, tight junction proteins claudin-1, occluding, and ZO-1 were absent from the cell-cell contact sites but were present in the cytoplasm [78]. Inhibition of MEK1 activity recruited all three proteins to the cell membrane leading to a restoration of the tight junction barrier function in MDCK cells [78]. However, in yet another study though using breast cancer cells, MEK1 inhibition neither affected the mRNA or protein levels of claudin-1, occludin and/or ZO-1 nor altered the subcellular cytoplasmic distribution of claudin-1 to be more membrane specific [79]. Further, studies have implicated protein kinase C in the regulation of TJs through phorbol ester stimulation [80, 81]. Also, PKA-dependent regulation of TJs was recently demonstrated. Claudin-3 and -4 can be phosphorylated in ovarian cancer cells by PKA, a kinase frequently activated in ovarian cancer [82] (Figure 3). Furthermore, modulation of MAP Kinase signaling specifically ERK 1/2 and P-P38 as well as PI-3 Kinase have profound effect upon tight junction sealing and claudin expression [83]. Similarly, lysine deficient protein kinase 4 (WNK4) can phosphorylate multiple claudins and increase paracellular permeability [84]. Most claudin proteins have putative serine and/or threonine phosphorylation sites in their cytoplasmic carboxy-terminal domains. The consequences of the differential modulation brought about by these kinases on these claudins remain to be determined but may contribute to ovarian tumorigenesis. 

Growth Factor receptors that are important in the regulation of cell proliferation and survival including EGF, HGF and IGF receptors regulate claudin expression and cellular distribution though once again in cell/tissue specific manner [85–88]. In addition, recent studies related to intestinal inflammation have suggested roles of cytokines including TNF-*α*, INF-*γ*, IL-13 in the regulation of claudins expression [89]. 

Endocytic recycling of claudin proteins is also a potential mechanism of claudin regulation [90], and palmitoylation [91] of these proteins has also been found to influence claudin protein stability. At the transcriptional level, transcription factors such as Snail [92], Cdx-2, HNF-*α*, and GATA-4 [93, 94] can bind to the promoter regions of various claudin genes and affect their expression. Furthermore, we have shown that colonic claudin-1 transcripts are regulated by Smad-4, a known tumor suppressor as well as HDAC inhibitors and thus support a complex regulation at multiple levels [95, 96].

## 5. Conclusion

Irrespective of the diverse source of cancer growth and/or heterogeneity among cancer patients regarding the cancer originated from the same tissue source, it is well accepted that Epithelial to Mesenchymal Transition (EMT) is a cellular event central to the initiation and progression of tumorigenesis. This raises the question: what these diverse cancers have in common? Importantly, majority of cancer-related deaths result from the cancers of epithelial origin and include cancers of the colon, prostate, bladder, lung, esophagus, breast, pancreas, ovary, and liver. Although their differentiated properties vary, they are all composed principally of epithelial cells which share similar basic features including polarity and barrier function. So, the question arises: what underlie the differential properties and/or response to the cancer therapy between cancers originated from different epithelial organs irrespective of the similarities among their basic building units and their properties? Cell-cell adhesion weakens or is lost during the process of EMT or as dedifferentiation of epithelial cells. A critical role of E-cadherin, principal constituent of adherens junction, in the regulation of EMT is known, however it does not help understand the diversity/heterogeneity among the cancers of epithelial origin. Importantly, claudins are expressed in the epithelial cells and in a tissue-specific manner and changes among claudin family members in cancer follow tissue-specific and sometimes contrasting pattern. Thus, claudin family of proteins may hold the potential cue to the heterogeneity among the tumors of epithelial origin and beyond being useful markers may also help provide therapeutic opportunities suited for specific cancer type.

## Figures and Tables

**Table 1:** Expression of Claudins in Cancer.

Type of Malignancy	Claudin gene		Expression
Breast Carcinoma	CLDN1		Down
	CLDN3		Up
	CLDN4		Up
	CLDN7		Down
Biliary tract Carcinoma	CLDN4		Up
Colorectal Carcinoma	CLDN1		Up
	CLDN8		Down
	CLDN12		Up
Endometrial endometrioid Carcinoma	CLDN1		Down
	CLDN2		Up
Endometrial seropapillary Carcinoma	CLDN1		Up
	CLDN2		Down
Gastric adenocarcinoma	CLDN1		Up
	CLDN3		Up
	CLDN4		Up
	CLDN5		Up
Hepatocellular carcinoma	CLDN4		Down
	CLDN7		Up
Hepatoblastoma (Fetal)	CLDN1		Up
	CLDN2		Up
	CLDN3		Down
	CLDN4		Down
	CLDN7		Down
Head & Neck (SCC)	CLDN7		Down
Lung cancer (Adenocarcinoma)	CLDN1		Down
	CLDN5		Up
Lung cancer (SCC)	CLDN1		Up
	CLDN5		Down
Meningioma	CLDN1		Up
Mesothelioma	CLDN4		Down
	CLDN5		Down
Metastatic Melanoma	CLDN1		Down
Oncocytoma	CLDN7		Down
	CLDN8		Up
Ovarian epithelial Carcinoma	CLDN1		Up
	CLDN3		Up
	CLDN4		Up
	CLDN5		Up
	CLDN7		Up
Ovarian sex cord stromal Tumors	CLDN1		Down
	CLDN3		Down
	CLDN4		Down
	CLDN5		Down
Pancreatic Carcinoma	CLDN1		Up
	CLDN4		Up
Pleura (metastatic adenocarcinoma)	CLDN3		UP
	CLDN4		UP
Prostate Carcinoma	CLDN1		Up
	CLDN2		Down
	CLDN3		Up
	CLDN4		Up
	CLDN5		Down
	CLDN7		Up
Renal cell Carcinoma	CLDN1		Up
	CLDN3		Up
	CLDN4		Up
Renal cell Carcinoma (Chromobhobe)	CLDN7		Up
Tongue (SCC)	CLDN1		Up
	CLDN4		Up
	CLDN7		Up
Thyroid Carcinomas	CLDN1		Up
	CLDN4		Up
	CLDN7		Up
Undifferentiated Thyroid Carcinoma	CLDN1		Down
